# Extended Sequence Typing of *Campylobacter* spp., United Kingdom 

**DOI:** 10.3201/eid1410.071109

**Published:** 2008-10

**Authors:** Kate E. Dingle, Noel D. McCarthy, Alison J. Cody, Tim E.A. Peto, Martin C. J. Maiden

**Affiliations:** John Radcliffe Hospital, Oxford, UK (K.E. Dingle, T.E.A. Peto); University of Oxford, Oxford (K.E. Dingle, N.D. McCarthy, A.J. Cody, T.E.A. Peto, M.C.J. Maiden)

**Keywords:** Campylobacter jejuni, Campylobacter coli, multilocus sequence typing, molecular epidemiology, antigen sequence typing, dispatch

## Abstract

Supplementing *Campylobacter* spp. multilocus sequence typing with nucleotide sequence typing of 3 antigen genes increased the discriminatory index achieved from 0.975 to 0.992 among 620 clinical isolates from Oxfordshire, United Kingdom. This enhanced typing scheme enabled identification of clusters and retained data required for long-range epidemiologic comparisons of isolates.

Human campylobacteriosis remains a global public health problem. Although many risk factors for this foodborne zoonotic disease are known, the relative contributions of different transmission routes are poorly quantified. Furthermore, the sources of particular infections are frequently obscure and outbreaks are rarely detected (*1*). The high genetic and antigenic diversity of the 2 major causes of campylobacteriosis, *Campylobacter jejuni* and *C*. *coli*, have proved to be obstacles in routine surveillance, outbreak identification, and source attribution.

Nucleotide sequence–based isolate characterization methods such as multilocus sequence typing (MLST) successfully catalog bacterial pathogens and provide a rational, definitive, and portable typing method with complete reproducibility among laboratories (*2*). Because many of the sequence types (STs) or their close relatives are observed on multiple occasions with wide geographic distribution, MLST is highly effective for long-range epidemiologic studies (*3*). However, this characteristic can limit the application of MLST to outbreak identification (*4*). We combined MLST data with sequence data of the short variable region (SVR) of the *flaA* and *flaB* loci, previously used to type *Campylobacter* spp./isolates (*5*,*6*), and a novel typing system based on *porA* locus encoding the variable outer membrane protein PorA. The resultant high-resolution 10-locus typing scheme was used to characterize 620 isolates obtained from 584 persons with human campylobacteriosis from September 2003 through September 2004 in Oxfordshire (population ≈600,000), United Kingdom; 36 isolates obtained by sampling the same patient more than once were used to confirm reproducibility.

## The Study

**Figure 1:**
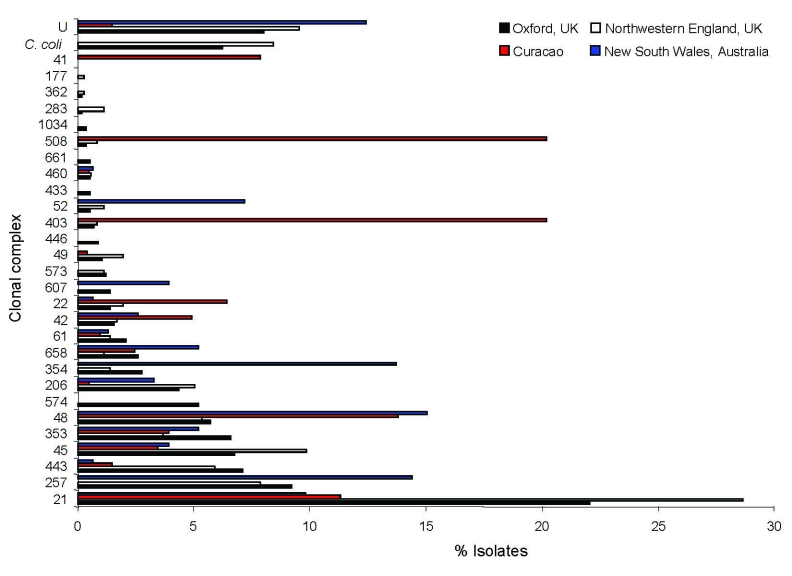
Relative abundance of clonal complexes of *Campylobacter* spp. detected in Oxfordshire, United Kingdom, during a 1-year study compared with clonal complexes detected in 3 other studies of human *Campylobacter* spp. infections in northwestern England (*7*), New South Wales, Australia (*8*), and Curaçao (*9*).

A comparison of our results with published population-based 7-locus MLST studies showed that the relative abundance of different clonal complexes in northwestern England from April 2003 through March 2004 was similar (*7*), presumably reflecting widely distributed foods in the United Kingdom. An exception was ST-574 complex, the central genotype, which represented >5% of cases in Oxfordshire but was absent from northwestern England. The clonal complex distribution of 171 isolates collected in New South Wales, Australia (*8*), was distinct from the 2 English datasets, although many clonal complexes were present in all 3 datasets. Fewer clonal complexes, with different relative abundances, were present in a dataset from Curaçao (*9*), likely because of different infection sources in the Dutch West Indies (Figure 1).

The differences in relative abundance of clonal complexes were mirrored by *F_ST_* values calculated from concatenated nucleotide sequences of the MLST loci, which indicated that the Australian dataset was 3.6% differentiated and the Curaçao set 9.9% differentiated from the Oxfordshire dataset. One clonal complex detected in Curaçao was absent in the United Kingdom (ST-41 complex) and 13 of the clonal complexes detected in the United Kingdom were absent in Curaçao.

The antigen loci added further resolution to the Oxfordshire dataset: 575 (98.6% of isolates) *flaA* SVR sequences contained 130 distinct SVR alleles, and 567 (97.1%) *flaB* sequences contained 111 SVR alleles. The allele fragment ≈630bp used for *porA* typing was amplified and sequenced with primers MOMP-1 (5′-GAT GGT TTA ACT CTA GCT GC-3′) or MOMP-3 (5′-GAT GGT TTA GTW GGM ACA GG-3′) and MOMP-2 (5′-TGA GAA GTT AAG TTT TGG AG AG-3′). *PorA* allele and variant numbers were assigned and sequences were deposited in a database (http://hercules.medawar.ox.ac.uk/momp). A description of amplification conditions is also available at this website. Of the 558 (95.5% of the isolates) *porA* sequences assigned, 1 occurred in 65 isolates whereas 135 alleles occurred only once.

The discriminatory index (DI) (*10*) was calculated for various subsets of these data. Each additional antigen gene increased discrimination relative to MLST data alone. The combination of *flaA* SVR and *flaB* SVR (DI = 0.976) provide a similar level of discrimination as *porA* (DI = 0.972). The DI obtained with the *porA* gene fragment alone was similar to that obtained previously with a larger fragment of the same gene (DI = 0.973) (*11*). The 10-locus combination provided a degree of discrimination (DI = 0.992) higher than those published for pulsed-field gel electrophoresis fingerprinting, antigen typing, or MLST (*4*,*6*,*9*,*12*,*13*). These studies calculated DI, ignoring the fact that some isolates probably shared a common source and may have underestimated the true DI, the capacity to discriminate between epidemiologically unrelated isolates. For the same reason, our study is also likely to have underestimated the true DI.

**Figure 2:**
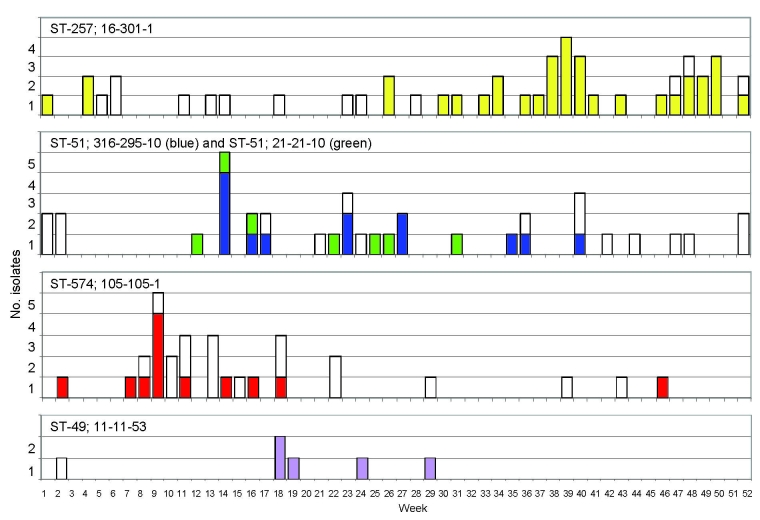
Clusters of related 10-locus types of *Campylobacter* spp. detected in Oxfordshire, United Kingdom, during a 1-year study. Five groups of isolates with identical genotypes show statistically significant clustering in time (p values are shown in the Table). Each group is indicated by 1 color. White bars indicate other isolates that share the same sequence type (ST) but that are differentiated by their different antigen type. Numbers of isolates of each genotype are shown on a weekly basis; week 1 corresponds to the start of the study on September 15, 2003.

There were 68 groups of >2 isolates with identical 10-locus types, ranging in size from 2 to 34 isolates and accounting for 283 (48.5%) of the independent isolates. Of the remaining isolates, 290 (49.7%) had unique types, typing data were incomplete for the remaining 10 isolates (1.7%). A permutation test with 283 isolates belonging to a cluster showed highly significant temporal clustering of *Campylobacter* spp. isolates of identical genotype (p<0.0001). The extent of clustering was independent of group size (data not shown). Of 16 groups of >5 identical isolates, 5 exhibited significant temporal clustering (Table, Figure 2). Isolates belonging to the largest of these groups, comprising 34 isolates of ST-257, *flaA* 16, *flaB* 301, and *porA* 1, were submitted mainly in the last part of the study year with a peak of 5 isolates in week 39 (Figure 2). The second largest group, comprising 13 isolates, shared ST-51 and an identical *porA* type with a genotypic group comprising 7 isolates, but the 2 groups were distinct at both *flaA* and *flaB* loci. All members of these 2 groups were isolated from week 14 through week 40. Of the 12 isolates comprising the third largest group, all but 1 were isolated over a 17-week period (week 2 through week 18) at the beginning of the year (Figure 2). The smallest group of isolates to show evidence for temporal clustering comprised 5 identical isolates obtained during weeks 18–29. Some of the other genotypic groups were seen throughout the year, with no evidence for temporal clustering; for others, weak evidence of clustering was found (Table).

**Table:** Temporal association of genotypically identical isolates of *Campylobacter* spp., United Kingdom*

ST	10-locus genotype			No. isolates	p value of temporal association
	*flaA* SVR	*flab* SVR	*porA*		
49	11	11	53	5	0.0005
206	14	96	7	5	0.36
583	239	177	43	5	0.85
45	8	8	44	6	0.92
48	32	103	14	6	0.44
354	18	18	57	6	0.42
51	21	21	10	7	0.0007
475	105	105	67	7	0.16
50	36	36	6	8	0.35
827	255	236	33	8	0.49
19	36	36	7	9	0.15
658	5	5	25	9	0.19
104	36	36	14	11	0.14
574	105	105	1	12	<0.0001
51	316	295	10	13	<0.0001
257	16	301	1	34	<0.0001

*ST, sequence type; SVR, short variable region.

## Conclusions

Clusters were detected by this method but represented a small part of the overall disease incidence (*14*). Temporal association within these clusters suggests that they may represent undetected outbreaks. Further epidemiologic information unavailable to this study, which was based solely on laboratory isolates, would be needed to confirm or refute this possibility.

The clusters occurred over periods longer than the typical duration of outbreaks of gastroenteritis, which was consistent with episodes of contamination entering the food chain rather than single proximate-point source events. These findings indicated that many of these clusters may be associated with widely distributed foods. The observed temporal association of groups of identical isolates could also be caused by certain genotypes having different seasonality or environmental sources from sporadic cases. In either scenario, the data supported the interpretation that cases sharing an identical genotype were more likely to be epidemiologically linked than those infected with different genotypes.

In conclusion, the 10-locus typing scheme is highly discriminatory for *C*. *jejuni* and *C. coli* isolates and provides information that can be used flexibly to support long-range comparisons and short-term epidemiology. The scheme can be applied in real time or near real time, enabling the data to be used to identify outbreaks and inform public health interventions. When combined with improved genetic methods of attributing source of bacterial isolates (*15*), this approach will contribute to refining the epidemiology of these enigmatic pathogens.

## References

[1] Humphrey T, O’Brien S, Madsen M. Campylobacters as zoonotic pathogens: a food production perspective. Int J Food Microbiol. 2007;117:237–57. 10.1016/j.ijfoodmicro.2007.01.006 17368847

[2] Dingle KE, Colles FM, Wareing DRA, Ure R, Fox AJ, Bolton FJ, Multilocus sequence typing system for *Campylobacter jejuni.* J Clin Microbiol. 2001;39:14–23. 10.1128/JCM.39.1.14-23.2001 11136741 PMC87672

[3] Dingle KE, Colles FM, Ure R, Wagenaar J, Duim B, Bolton FJ, Molecular characterization of *Campylobacter jejuni* clones: a rational basis for epidemiological investigations. Emerg Infect Dis. 2002;8:949–55. 12194772 10.3201/eid0809.02-0122 PMC2732546

[4] Sails AD, Swaminathan B, Fields PI. Utility of multilocus sequence typing as an epidemiological tool for investigation of outbreaks of gastroenteritis caused by *Campylobacter jejuni.* J Clin Microbiol. 2003;41:4733–9. 10.1128/JCM.41.10.4733-4739.2003 14532212 PMC254344

[5] Meinersmann RJ, Helsel LO, Fields PI, Hiett KL. Discrimination of *Campylobacter jejuni* isolates by *fla* gene sequencing. J Clin Microbiol. 1997;35:2810–4. 9350739 10.1128/jcm.35.11.2810-2814.1997 PMC230067

[6] Mellmann A, Mosters J, Bartelt E, Roggentin P, Ammon A, Friedrich AW, Sequence-based typing of *flaB* is a more stable screening tool than typing of *flaA* for monitoring of *Campylobacter* populations. J Clin Microbiol. 2004;42:4840–2. 10.1128/JCM.42.10.4840-4842.2004 15472357 PMC522316

[7] Sopwith W, Birtles A, Matthews M, Fox A, Gee S, Painter M, *Campylobacter jejuni* multilocus sequence types in humans, northwest England, 2003–2004. Emerg Infect Dis. 2006;12:1500–7. 17176563 10.3201/eid1210.060048 PMC3290937

[8] Mickan L, Doyle R, Valcanis M, Dingle KE, Unicomb L, Lanser J. Multilocus sequence typing of *Campylobacter jejuni* isolates from New South Wales, Australia. J Appl Microbiol. 2007;102:144–52. 10.1111/j.1365-2672.2006.03049.x 17184329

[9] Duim B, Godschalk PC, van den Braak N, Dingle KE, Dijkstra JR, Leyde E, Molecular evidence for dissemination of unique *Campylobacter jejuni* clones in Curacao, Netherlands Antilles. J Clin Microbiol. 2003;41:5593–7. 10.1128/JCM.41.12.5593-5597.2003 14662946 PMC309031

[10] Hunter PR, Gaston MA. Numerical index of discriminatory ability of typing systems: an application of Simpson’s index of diversity. J Clin Microbiol. 1988;26:2465–6. 3069867 10.1128/jcm.26.11.2465-2466.1988 PMC266921

[11] Huang S, Luangtongkum T, Morishita TY, Zhang Q. Molecular typing of *Campylobacter* strains using the *cmp* gene encoding the major outer membrane protein. Foodborne Pathog Dis. 2005;2:12–23. 10.1089/fpd.2005.2.12 15992295

[12] Schouls LM, Reulen S, Duim B, Wagenaar JA, Willems RJ, Dingle KE, Comparative genotyping of *Campylobacter jejuni* by amplified fragment length polymorphism, multilocus sequence typing, and short repeat sequencing: strain diversity, host range, and recombination. J Clin Microbiol. 2003;41:15–26. 10.1128/JCM.41.1.15-26.2003 12517820 PMC149617

[13] Thakur S, Morrow WE, Funk JA, Bahnson PB, Gebreyes WA. Molecular epidemiologic investigation of *Campylobacter coli* in swine production systems, using multilocus sequence typing. Appl Environ Microbiol. 2006;72:5666–9. 10.1128/AEM.00658-06 16885327 PMC1538767

[14] Pebody RG, Ryan MJ, Wall PG. Outbreaks of campylobacter infection: rare events for a common pathogen. Commun Dis Rep CDR Rev. 1997;7:R33–7. 9080726

[15] McCarthy ND, Colles FM, Dingle KE, Bagnall MC, Manning G, Maiden MC, Host-associated genetic import in *Campylobacter jejuni.* Emerg Infect Dis. 2007;13:267–72. 17479890 10.3201/eid1302.060620 PMC2063414

